# Genome-wide analysis of ATP-binding cassette transporter provides insight to genes related to bioactive metabolite transportation in *Salvia miltiorrhiza*

**DOI:** 10.1186/s12864-021-07623-0

**Published:** 2021-05-01

**Authors:** Li Yan, Jianhong Zhang, Hongyu Chen, Hongmei Luo

**Affiliations:** Engineering Research Center of Chinese Medicine Resource, Ministry of Education, Institute of Medicinal Plant Development, Chinese Academy of Medical Sciences & Peking Union Medical College, Beijing, China

**Keywords:** *Salvia miltiorrhiza*, Transporters, ATP-binding cassette (ABC) transporters, Gene family analysis, Tanshinone and salvianolic acid transport

## Abstract

**Background:**

ATP-binding cassette (ABC) transporters have been found to play important roles in metabolic transport in plant cells, influencing subcellular compartmentalisation and tissue distribution of these metabolic compounds. *Salvia miltiorrhiza* Bunge, known as Danshen in traditional Chinese medicine, is a highly valued medicinal plant used to treat cardiovascular and cerebrovascular diseases. The dry roots and rhizomes of *S. miltiorrhiza* contain biologically active secondary metabolites of tanshinone and salvianolic acid. Given an assembled and annotated genome and a set of transcriptome data of *S. miltiorrhiza*, we analysed and identified the candidate genes that likely involved in the bioactive metabolite transportation of this medicinal plant, starting with the members of the ABC transporter family.

**Results:**

A total of 114 genes encoding ABC transporters were identified in the genome of *S. miltiorrhiza*. All of these ABC genes were divided into eight subfamilies: 3ABCA, 31ABCB, 14ABCC, 2ABCD, 1ABCE, 7ABCF, 46ABCG, and 10 ABCI. Gene expression analysis revealed tissue-specific expression profiles of these ABC transporters. In particular, we found 18 highly expressed transporters in the roots of *S. miltiorrhiza*, which might be involved in transporting the bioactive compounds of this medicinal plant. We further investigated the co-expression profiling of these 18 genes with key enzyme genes involved in tanshinone and salvianolic acid biosynthetic pathways using quantitative reverse transcription polymerase chain reaction (RT-qPCR). From this RT-qPCR validation, we found that three ABC genes (*SmABCG46*, *SmABCG40*, and *SmABCG4*) and another gene (*SmABCC1*) co-expressed with the key biosynthetic enzymes of these two compounds, respectively, and thus might be involved in tanshinone and salvianolic acid transport in root cells. In addition, we predicted the biological functions of *S. miltiorrhiza* ABC transporters using phylogenetic relationships and analysis of the transcriptome to find biological functions.

**Conclusions:**

Here, we present the first systematic analysis of ABC transporters in *S. miltiorrhiza* and predict candidate transporters involved in bioactive compound transportation in this important medicinal plant. Using genome-wide identification, transcriptome profile analysis, and phylogenetic relationships, this research provides a new perspective on the critical functions of ABC transporters in *S. miltiorrhiza.*

**Supplementary Information:**

The online version contains supplementary material available at 10.1186/s12864-021-07623-0.

## Background

*Salvia miltiorrhiza* is a common medicinal plant used to treat inflammation and cardiovascular diseases because of its high quantities of biologically active hydrophilic salvianolic acid (SA) and lipophilic diterpenoids (tanshinones) in its roots or rhizomes [1]. *S. miltiorrhiza* is an ideal model medicinal plant for studying secondary metabolic biosynthesis. GGPP is the biosynthetic precursor of tanshinone, which is catalysed by copalyl diphosphate synthase (CPS) to form copalyl diphosphate. Then a series of cytochrome P450 monooxygenases (CYP450s) catalyses downstream oxidation reactions. Ferruginol, the catalytic product of CYP76AH1, is an important intermediate product in the biosynthetic pathway of tanshinone [2]. CYP76AH3 and CYP76AK1 are responsible for the conversion of ferruginol into intermediate compounds 11,20-dihydroxy ferruginol and 11,20-dihydroxy sugiol en route to becoming tanshinones [3]. SA biosynthesis is derived from 4-coumaroyl-3′,4′-dihydroxyphenyllactic acid (4C-DHPL), which is a combination of 3,4-dihydroxyphenyllactic acid (DHPL) and 4-coumaroyl-CoA. These two compounds are coupled by rosmarinic acid synthase (SmRAS) [4]. The 3-hydroxyl group is introduced by a cytochrome P450-dependent monooxygenase (SmCYP98A14) to form rosmarinic acid [4]. Significant progress has been made in the understanding of the biosynthetic pathways of these active ingredients in *S. miltiorrhiza*, but the transport and storage mechanisms of these compounds in plant cells have not yet been elucidated.

ATP-binding cassette (ABC) transporters, one of the few gene families present in all domains of life, are involved in a wide range of biological processes and play key roles in the transmembrane transport of metabolites across biological membranes by hydrolysing ATP in plant cells [5]. In most cases, the core functional unit of ABC transporters usually consists of a combination of two transmembrane domains (TMDs) and two nucleotide-binding domains (NBDs). The TMDs, which typically contain several (usually four to six) transmembrane hydrophobic alpha-helices, form a membrane-spanning pore which is involved in substrate recognition and solute movement across the phospholipid bilayer. The two NBDs couple ATP hydrolysis and ADP release to provide the driving force for transport. These NBDs contain several key conserved motifs: Walker A (G*X*_4_GK(ST)), Walker B ((RK)*X*_3_G*X*_3_L(hydrophobic)_3_), ABC signature, Q-loop, D-loop and H-loop [6–8]. In general, ‘full-sized’ ABC proteins are comprised of two pairs of TMD–NBD and fully function as transporters, while ‘half-sized’ ABC proteins have only one TMD–NBD that must form homo- or heterodimers to become a transporter [7].

Genome analyses of model plants (e.g. *Arabidopsis* and rice) show that the plant genome contains a large number of ABC transporters compared to animals and other eukaryotes [6]. This increase of ABC genes has significantly improved the ability of plants to adapt to various environmental stressors [7]. Usually located in plant cell plasma membranes, vacuole membranes, and other organelle membranes, ABC transporters regulate a membrane’s absorption and efflux of specific substances such as secondary metabolites, sugars, amino acids, plant hormones, lipids, and metal ions [9, 10]. Because ABC proteins have a wide range of biochemical and physiological functions, are key to the transport of diverse substances, and thus important to disease resistance and detoxification, these proteins are essential to maintaining plant life [7].

The subfamily classification of plant ABC transporters is structured according to the subfamily nomenclature proposed by the Human Genome Organization [11]. This nomenclature is based on the phylogenetic relationships of NBD amino acid sequences. Therefore, the eukaryotic ABC transporter family is divided into eight subfamilies: ABCA, ABCB, ABCC, ABCD, ADCE, ABCF, ABCG and ABCH [11, 12]. However, no ABCH subfamily is found in plants; rather ABCH is replaced by ABCI, which exists in plants but is absent in animals. The division of these subfamilies is based on the phylogenetic relationships of the NBD amino acid sequences and is also largely supported by domain organization (the order of domains in the ABC protein), although some examples of subfamilies include both full-sized and half-sized transporters [11]. In plants, the best-identified subfamilies of ABC proteins are multidrug resistance (MDR), MRP, PDR, and white–brown complex homologue (WBC) [10]. The *Arabidopsis* ABC protein superfamily consists of full-sized transporters, half-sized transporters, and soluble proteins [10]. The full-sized transporters include the MDRs, MRPs, PDRs, peroxisomal membrane proteins (PMPs), and ABC one homologues (AOHs). The half-sized transporters include PMPs, WBCs, ABC two homologues (ATHs), ABC transporter of the mitochondrions (ATMs), and transporters associated with antigen processing (TAPs). The soluble proteins include 2′,5′-oligoadenylate activated RNase inhibitor homologues (RLIs), yeast general control non-repressible homologues (GCNs) and structural maintenance of chromosome homologues (SMCs) [10]. In contrast, the non-intrinsic ABC protein (NAP) subfamily cannot be classified in this way because NAPs are a heterogeneous group of soluble or non-intrinsic membrane proteins [10].

A genome-wide analysis enables the classification of the ABC subfamilies on the basis of genomic information. This genetic approach may reveal information about evolutionary processes and the diversity and relationships of ABC genes and their proteins, thus serving as a basic resource for predicting more functions and detecting the relationship between genes and evolutionary diversity of different species. Complete inventories of plant ABC transporters are available for *Arabidopsis* [6], *Oryza sativa* [12], *Vitis vinifera* [13], *Zea mays* [14], *Brassica napus* [15], *Ananas comosus* [16], *Solanum lycopersicum* [17], *Capsicum annuum* [18], *Hevea brasiliensis* [19] and *Lotus japonicas* [20]. The recent sequencing of *S. miltiorrhiza* whole genome and the large published set of transcriptome leads to our analysis of ABC transporters on a genome scale [21–25].

Here, we describe the first complete analysis of the ABC transporter superfamily in the *S. miltiorrhiza* genome. A total of 114 genes, divided into eight subfamilies, were annotated to encode for ABC transporter proteins in *S. miltiorrhiza*. We characterized all of the ABC proteins in *S. miltiorrhiza* and included them a phylogenetic analysis with the ABC proteins from *Arabidopsis* and other plants. On the basis of the co-expression analysis of key enzyme genes involved in the biosynthetic pathways of the active ingredients in *S. miltiorrhiza*, we predicted that three ABCG and one ABCC subfamily ABC transporter genes were involved in the transport of the bioactive metabolites tanshinone and SA, respectively. In addition, the ABC proteins involved in the transport of plant hormones, secondary metabolites, ions, and other substances were predicted in *S. miltiorrhiza*.

## Results

### Identification of ABC transporters in the *S. miltiorrhiza* genome

A total of 204 homologous ABC transporters were annotated in the *S. miltiorrhiza* genome on the basis of sequence alignment with all of the ABC transporters in the *Arabidopsis* TAIR11 database (Araport11 genome release). These 204 ABC transporters in *S. miltiorrhiza* (SmABCs) were verified by manually confirming the integrity of the conserved domains and motifs of ABC proteins. Ultimately, 114 genes encoding for ABC transporters were identified in the *S. miltiorrhiza* genome (Table 1). Considering that a typical full-sized ABC protein contains at least 1200 amino acid residues [6] and that these 114 ABC transporters ranged in length from 186 to 1978 amino acid residue (Table 1), some of these shorter sequences might be pseudogenes or not full-length ABC transporters. Thirty-three SmABC protein sequences were shorter than their *Arabadopsis* homologous genes by at least 100 amino acids, including 14 genes from the ABCB subfamily, 3 genes from the ABCC subfamily, 13 genes from the ABCG subfamily; and 1 gene from each of the subfamilies ABCA, ABCD and ABCF, respectively (Table 1). These 33 SmABC genes may be partial sequences, not pseudogenes, and that they are shorter as a result of the limited quality and integrity of the available assembled genome of *S. miltiorrhiza*. Of the 114 identified SmABC transporters, 86 were intrinsic membrane proteins with TMDs. Of these 86 intrinsic membrane proteins, 50 were putative full-sized ABC transporters containing at least two TMD and two NBD domains, which were distributed in ABCB, ABCG and ABCC subfamilies (Table 1). Of the other 36 intrinsic membrane proteins, 31 were half-sized ABC transporters with one TMD and one NBD domain, and they were primarily distributed in the ABCF, ABCG and ABCI subfamilies (Table 1). The remaining 5 SmABC transporters were non-integrated proteins harbouring two TMD domains and one NBD domain or two NBD domains and one TMD domain, most of which were from the ABCB and ABCG subfamilies (Table 1). In addition, the remaining 28 genes were identified as non-intrinsic proteins, which encoded for proteins lacking TMD. Eighteen of these non-intrinsic proteins were grouped into five subfamilies (ABCB, ABCD, ABCE, ABCF and ABCG), and 10 of the proteins were divided into the ABCI subfamily (Table 1).

**Table 1:**
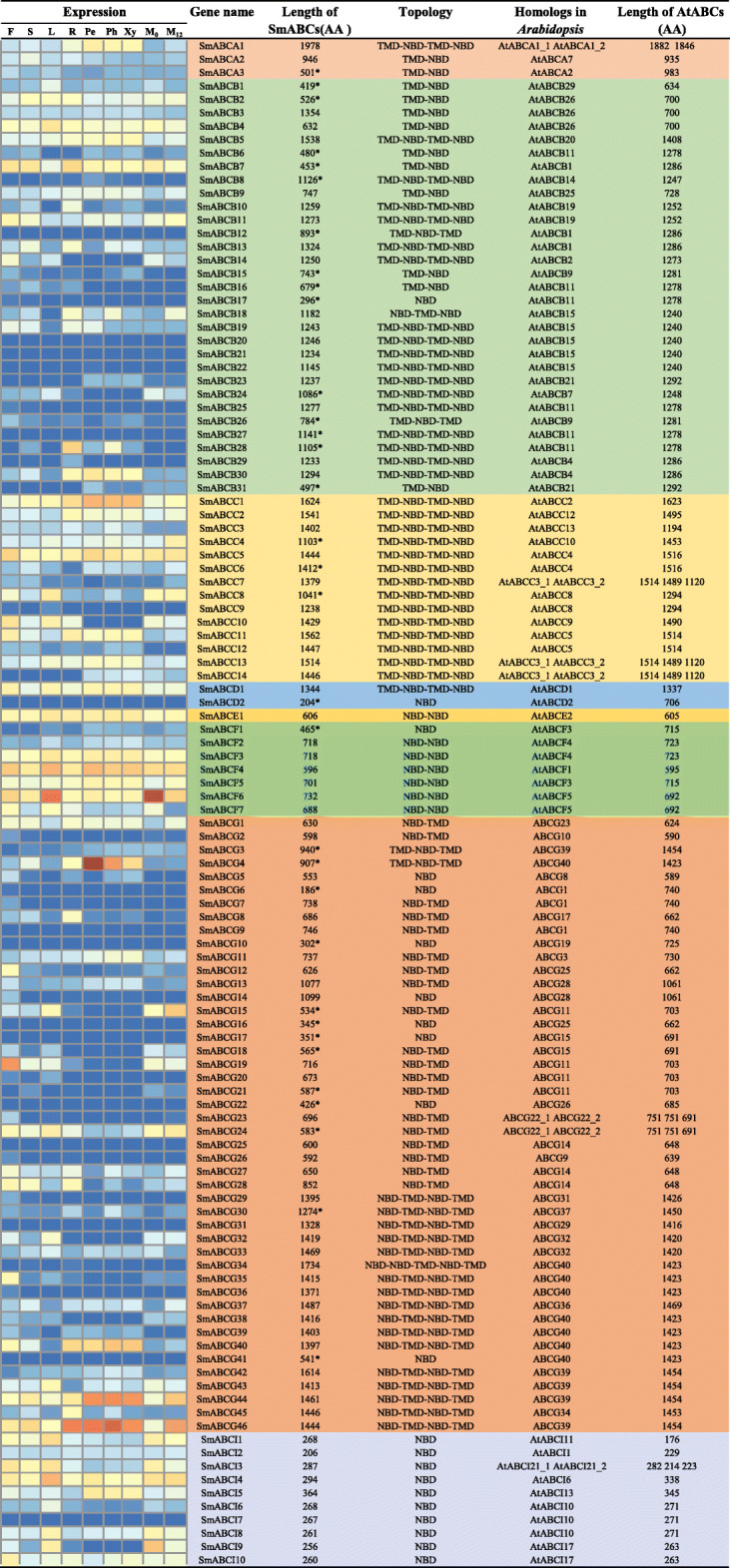
Inventory of ABC transporters of *S. miltiorrhiza* with the gene expression profiles

The relative gene expression levels of these *SmABCs* in different organs/tissues of *S. miltiorrhiza* were represented by color scales from red to yellow and from yellow to blue, indicating the order of gene expression levels from high to low. The organs/tissues used to detect gene expression levels include flowers (F), stems (S), leaves (L), roots (R), pericytes (Pe), phloem (Ph), and xylem (Xy). M_0_ represents the control leaves treated with MeJA for 0 h, and M_12_ represents the leaves treated with MeJA (200 μM) for 12 h. All expression data were derived from transcriptome data in our previous studies [23, 24]. NBD: nucleotide binding domain, TMD: transmembrane domain. The superscript “*” of the sequence length of SmABCs indicates that the length of the ABC proteins are shorter than theirs homologous gene of Arabidopsis at least 100 amino acids

Fifteen motifs of SmABC transporters were predicted and identified using the MEME (http://meme-suite.org/) which characterizes the diversity of ABC proteins (Additional file 1: Figure S1). These results showed that the conserved motifs amongst the SmABC proteins were similar. For example, the motifs of ABC signatures, Walker A and Walker B were present in these proteins (Additional file 1: Figure S1). The integrity of the full-sized transporter was verified by analyzing the arrangement of these three motifs in the ABC transporters. The lengths of the conserved motifs ranged from 20 to 50 amino acids. Additionally, the number of conserved motifs in each SmABC transporter ranged from 1 to 13 (Additional file 1: Figure S1). Moreover, the motifs of the ABC proteins belonging to the same subfamily were distributed in the same position. The ABC proteins with high similarity had the same motif and gene structure, whereas ABC proteins containing different motifs usually had different gene functions.

### Phylogenetic analysis of ABC transporters in *S. miltiorrhiza*

**Fig. 1:**
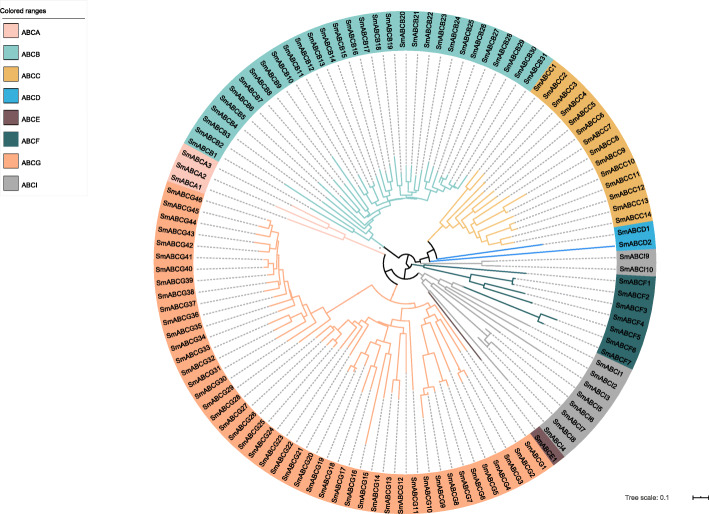
The phylogenetic analysis of SmABCs. Phylogenetic analysis was performed using the identified NBD amino acid sequence of 114 ABC protein in *S. miltiorrhiza*. The ClustalW program was used to align the amino sequence of all NBDs of the SmABCs, and the phylogenetic analysis was performed. The NJ tree was constructed from the protein sequences of SmABCs using MEGA6 with 1000 bootstrap copies. The Human Genome Organization (HUGO) nomenclature was used to name all the SmABCs. The ABCI subfamily of *S. miltiorrhiza* was not clustered similar to the ABCA-ABCG subfamilies

Phylogenetic analysis was used to classify SmABC transporters into the subfamilies. The 114 SmABC transporters were divided into eight subfamilies: 3 in ABCA, 31 in ABCB, 14 in ABCC, 2 in ABCD, 1 in ABCE, 7 in ABCF, 46 in ABCG and 10 in ABCI (Fig. 1). The distribution of the SmABC subfamilies was similar to that of other plants, and the ABCG subfamily had significantly higher number of genes compared to the other subfamilies. A phylogenetic tree was constructed using both the SmABC transporter identified in this study and ABC proteins identified in other plants to infer the function and evolutionary relationships of the transporters in *S. miltiorrhiza.* All ABC proteins used in this analysis are listed in Additional file 2: Table S1.

### Analysis of ABC transporter subfimilies in *S. miltiorrhiza*

#### ABCA subfamily

The plant ABCA subfamily includes one full-sized and several half-sizedABC proteins. In *Arabidopsis*, AtABCA1 is the only full-sized ABCA transporter and is the largest ABC protein consisting of 1882 amino acid residues with domains arranged in a forward direction (TMD1-NBD1-TMD2-NBD2) [6, 12]. The domains of half-sized transporters of ABCA subfamily arranges in a forward direction as well (TMD1-NBD1). To data, these transporters have only been found in plants and prokaryotes [26, 27].

**Fig. 2:**
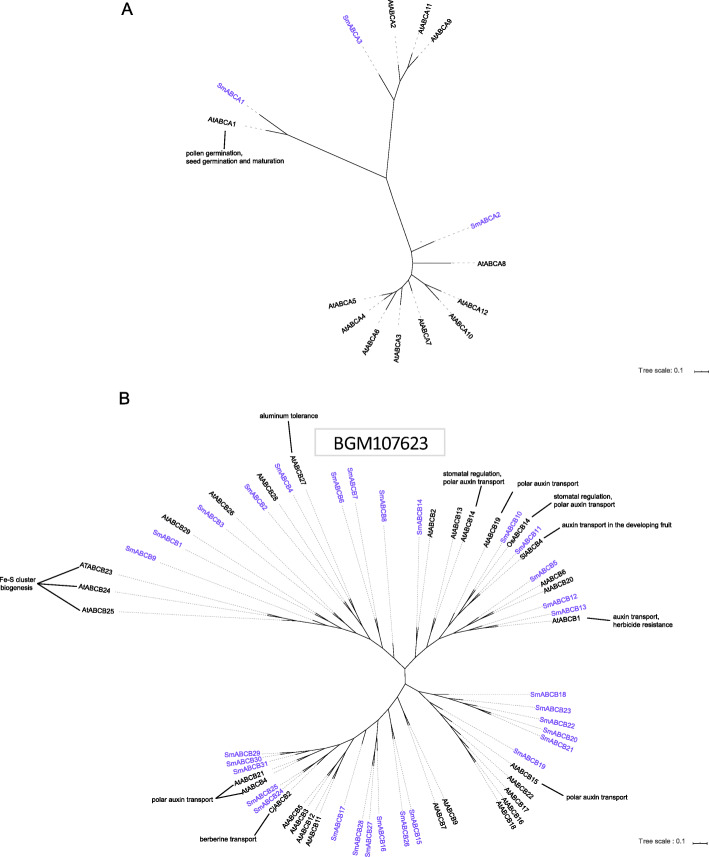
Phylogenetic tree of the ABCA and ABCB subfamily. Phylogenetic analysis of ABCA (**a**) and ABCB (**b**) proteins of *S. miltiorrhiza*, Arabidopsis and other plants

Three genes (*SmABCA1–3*) were annotated to be ABCAs in the *S. miltiorrhiza* genome (Fig. 2a). SmABCA1 was a full-sized ABCA transporter with high sequence homology to AtABCA1 (Table 1 and Fig. 2a). *SmABCA1* was also a larger ABC transporter in *S. miltiorrhiza*, consisting of 1978 amino acid residues. Compared to other plant tissues, *SmABCA1* was highly expressed in the roots of *S. miltiorrhiza* (Table 1), implying that *SmABCA1* might have an important function in the roots of *S. miltiorrhiza*. In contrast, SmABCA2 and SmABCA3 were half-sized transporters in the *S. miltiorrhiza* genome.

#### ABCB subfamily

The ABCB subfamily, the second largest ABC transporter subfamily, consists of both full-sized and half-sized transporters [7]. The domains of ABCB transporters are arranged in a forward direction (TMD1-NBD1-TMD2-NBD2). AtABCB1 was the first cloned and identified ABC transporter, playing roles in multiple herbicide tolerances in plants [28]. Full-sized ABCB proteins play an important role in bidirectional auxin transport [29], stomatal regulation [30], and metal tolerance in *Arabidopsis* [31], most of which are located in the plasma membrane of plants [32]. Half-sized ABCB transporters are involved in the biogenesis of Fe-S clusters in the mitochondria [33]. In this study, 31 genes were assigned to the ABCB subfamily in *S. miltiorrhiza*, 17 of which were full-sized transporters (Table 1 and Fig. 2b). These three SmABCB proteins, SmABCB10, SmABCB11, and SmABCB13, encoded for full-sized transporters and had sequence homology with *Arabidopsis* AtABCB1 [34] and AtABCB19 [35] (Fig. 2b) as well as OsABCB14 [36], and tomato SlABCB4 [37], all of which are involved in auxin transport. The expression profiles of these three transporter genes had no tissue specificity in *S. miltiorrhiza* (Table 1). *SmABCB30* was highly expressed in the roots of *S. miltiorrhiza*, particularly in the periderm (Table 1). The tissue-specific expression of *SmABCB30* was similar to that of the berberine transporter *CjABCB2* in *Coptis chinensis* [38], indicating that *SmABCB30* might be involved in the transport of secondary metabolites in *S. miltiorrhiza*. We also found that SmABCB29, SmABCB30 and SmABCB31 had sequence homology with AtABCB4 and AtABCB21 (Fig. 2b), and the latter two transporters are responsible for auxin transport in *Arabidopsis* [39, 40]. The full-sized transporter *SmABCB14* was highly expressed in the flowers, while *SmABCB28* and *SmABCB18* were actively expressed in the roots (Table 1). SmABCB19 clustered closely with AtABCB15, which is implicated in auxin transport of *Arabidopsis* [41]. The half-sized transporter SmABCB9 was particularly similar to AtABCB23, AtABCB24 and AtABCB25 in *Arabidopsis* (Fig. 2b). These three transporters in *Arabidopsis* are involved in the biogenesis of Fe/S clusters [33], and their expression is up-regulated after methyl jasmonate (MeJA) treatment, which was similar to the MeJA-induced expression profile of *SmABCB9*. The half-sized transporter *SmABCB4* was highly expressed in all plant organs (Table 1). SmABCB4 clustered closely with AtABCB27 (Fig. 2b), which is known to be involved in aluminium sequestration [31].

#### ABCC subfamily

ABCC subfamily consists of members which are at least 1500 amino acid residues in length, are only full-sized ABC transporters in *Arabidopsis* [10], and harbour an additional ABCC-specific hydrophobic N-terminal transmembrane domain (TMD0) [42]. The domains of the ABCC proteins were arranged in a forward direction (TMD0-TMD1-NBD1-TMD2-NBD2) [10]. Most ABCC transporters in plants are located in the vacuole membrane, and a few have been reported to reside on the plasma membrane [43, 44]. ABCC proteins are involved in heavy metal tolerance [45, 46], glutathione S-conjugate transport [47], and phytate storage in plants [44]. In addition, ABCCs are responsible for the transport of secondary metabolites in several plants. For example, ZmMRP3 is required for anthocyanin accumulation in maize [48] and VvABCC1is found to be involved in transport anthocyanins in grape [49], respectively; and CsABCC4a in saffron mediated crocin accumulation in cell vacuoles [50].

**Fig. 3:**
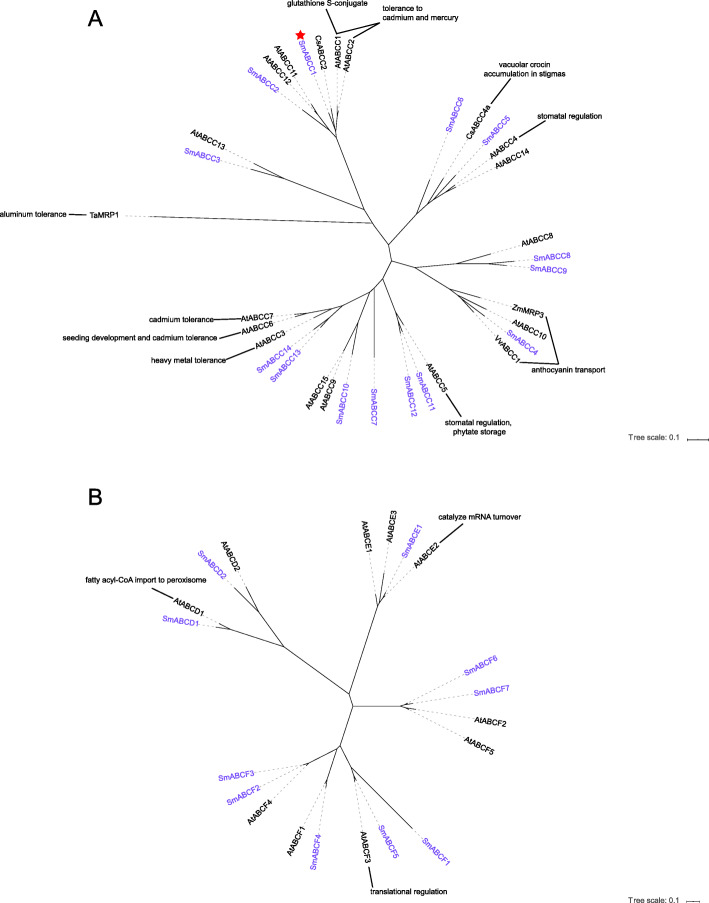
Phylogenetic tree of the ABCC and ABCD/E/F subfamily. Phylogenetic analysis of ABCC (**a**) and ABCD/E/F (**b**) proteins of *S. miltiorrhiza*, Arabidopsis and other plants

The transporter genes of the ABCC subfamily were expressed in all organs and tissues of *S. miltiorrhiza* (Table 1). *SmABCC2* and *SmABCC1* were expressed more highly in the roots of *S. miltiorrhiza* compared to other tissues (Table 1), and these two transporters were homologous to AtABCC11, AtABCC12, AtABCC1 and AtABCC2 in *A. thaliana* (Fig. 3a). *SmABCC5* was constitutively expressed in all organs (Table 1) and clustered with *Crocus sativus* CsABCC4a and *Arabidopsis* AtABCC4 (Fig. 3a). CsABCC4a is involved in the transport of crocin in *C. sativus* (saffron) [50] and AtABCC4 is responsible for transport of folic acid in *Arabidopsis* [51], respectively.

SmABCC4 was highly homologous to ZmMRP3 in maize [48] and VvABCC1 in grape [49], and the latter two transporters are related to anthocyanin accumulation and transport, respectively (Fig. 3a). Compared with other organs, the expression of *SmABCC4* in the leaves was higher under MeJA induction (Table 1), and this ABC transporter might be involved in the transport of secondary metabolites in *S. miltiorrhiza* leaves. SmABCC8 was located on another branch of the phylogenetic tree near SmABCC4 and was highly expressed in the leaves (Table 1), suggesting that *SmABCC8* might also participate in the transportation of substances in the leaves (Fig. 3a). *SmABCC11* was highly expressed in the flowers and roots, and its homologue AtABCC5 in *Arabidopsis* is related to the storage of phytate and loading of InsP6 in the seeds [44]. *SmABCC13* was highly expressed in the leaves and roots (Table 1) and clustered with *Arabidopsis* AtABCC6 and AtABCC3 (Fig. 3a), the latter two transporters are related to heavy metal tolerance [52, 53].

#### ABCD subfamily

The ABCD subfamily is located in the peroxisome membrane. In plants, this subfamily contains both full-sized and half-sized transporters. The full-sized transporter AtABCD1 in *Arabidopsis* is related to the import of long-chain fatty acyl-CoA into peroxisomes [54] and transport of 12-oxophytodienoic acid [55] and jasmonic acids [56]. Two ABCD members, SmABCD1 and SmABCD2, were found in the *S. miltiorrhiza* genome (Table 1 and Fig. 3b). *SmABCD1* was constitutively expressed in all organs and was homologous to AtABCD1 in *Arabidopsis* (Table 1 and Fig. 3b). We hypothesized that SmABCD1 had a similar function to AtABCD1 in *S. miltiorrhiza*.

#### ABCE and ABCF subfamilies

The ABCE subfamily, conserved in eukaryotes and archaea, consists of a soluble protein with only two conserved NBDs and without any detectable TMD. In *Arabidopsis*, AtABCE1 and AtABCE2 are involved in RNA interference (RNAi) regulation other than transport [57, 58]. AtABCE2 catalyzes the conversion of mRNA to DNA and participates in the biogenesis of the ribosome and in the initiation of translation in *Arabidopsis* [58]. ABCF similar to ABCE, is a soluble protein containing only two fused NBDs.

Only SmABCE1 was assigned to the ABCE subfamily in the *S. miltiorrhiza* genome, and it was constitutively expressed in all plant organs (Table 1 and Fig. 3b). Based on the functions of homologues AtABCE1 and AtABCE2 in *Arabidopsis*, SmABCE1 might play roles in the regulation of gene silencing. *S. miltiorrhiza* contained seven members of the ABCF subfamily, The four genes of *SmABCF3*/4/5/6 were highly expressed in all organs (Table 1). Amongst the members, *SmABCF6* was significantly expressed in high abundance in the leaves and was down-regulated after treatment with MeJA (Table 1). Considering that the homologues of SmABCF6 in yeast and humans are involved in the regulation of gene expression [59], *SmABCF6* might negatively regulate the expression of leaf tissue-specific genes under MeJA-induced conditions.

#### ABCG subfamily

The ABCG subfamily is the largest ABC protein subfamily in plants, including both full-sized and half-sized transporters. The NBD-TMD domains of this subfamily are arranged in opposite directions. Most of the characterised ABCGs are located in the plasma membrane [60, 61]. SpTUR2, one of the first identified transporter proteins in the ABCG subfamily, is involved in the transport of sclareol and herbicide resistance [62]. Moreover, transporters in the ABCG subfamily have been found to be related to the transport of paraquat, and may thereby modulate the tolerance of plants to herbicides [63]. ABCG transporters are widely involved in the transport of various compounds in plants [64, 65]. The ABCG proteins of *Arabidopsis* are involved in the transport of epidermal wax (AtABCG11) [66], plant hormones (ABA, IBA, cytokinin) [65], pathogen resistance [67] and kanamycin resistance [68]. Several ABCG proteins are also responsible for the synthesis of pollen walls (AtABCG1 and AtABCG16) [69], lignin biosynthesis [70], and exine formation on the pollen surface (AtABCG26) [71].

**Fig. 4:**
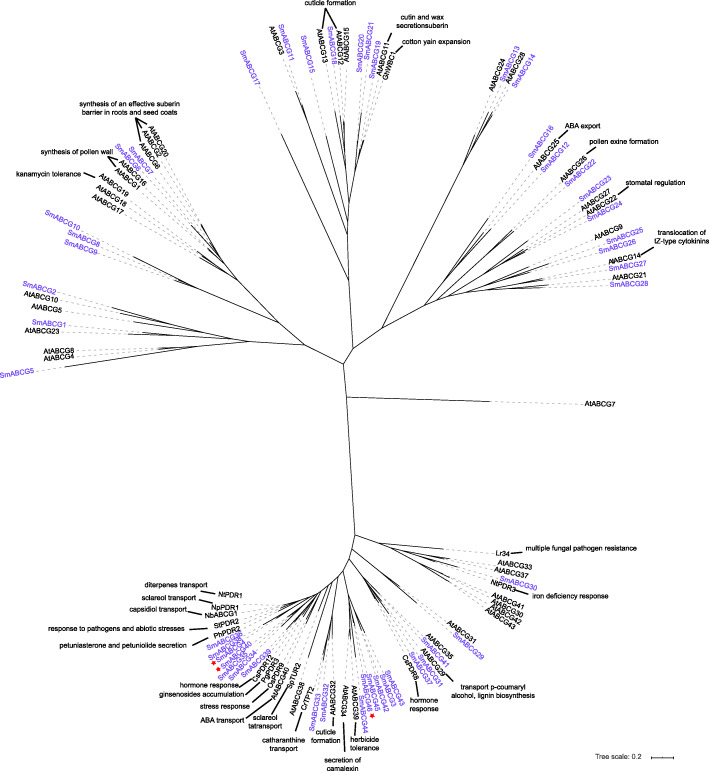
Phylogenetic tree of the ABCG subfamily. Phylogenetic analysis of ABCG proteins of *S. miltiorrhiza*, Arabidopsis and other plants

ABCG was also the largest subfamily of ABC transporters in *S. miltiorrhiza*, comprised of 46 members (Table 1 and Fig. 4). Four genes (*SmABCG40*, *SmABCG46*, *SmABCG4*, and *SmABCG44*) had tissue-specific expression profiles in this subfamily, all of which were highly expressed in the roots of *S. miltiorrhiza* (Table 1). Notably, *SmABCG4* was the most highly expressed gene in the periderm of *S. miltiorrhiza* roots (Table 1). Given that tanshinone is synthesized and accumulates in large amounts in the roots of *S. miltiorrhiza*, particularly in the periderm tissues [24], it is possible that these four transporters might be related to the transport of tanshinone in *S. miltiorrhiza*. Phylogenetic analysis revealed that SmABCG4 and SmABCG40 cluster relatively closely with the ginsenoside transporter *Panax ginseng* PgPDR3 [72] and the antifungal terpenoid transporter NpABC1 in *Nicotiana plumbaginifolia* and NtPDR1 in *N. tabacum* [73, 74] (Fig. 4). SmABCG46 and SmABCG44 were closely related to AtABCG39 [63] and AtABCG34 [64], which play roles in response to stress in *Arabidopsis*. MeJA induced the expression of *SmABCG46* and *SmABCG44* at different levels, which was homologous to the MeJA induction of *AtABCG34* in *Arabidopsis* (Table 1). Another full-sized transporter, *SmABCG45*, possessing the same gene structure and abundance as *SmABCG46*, was also highly expressed in the roots of *S. miltiorrhiza* (Additional file 1: Figure S1 and Table 1). These five genes of the SmABCG subfamily might be involved in terpenoid transport in *S. miltiorrhiza*, which might also mediate the stress responses of this medicinal plant. Although it has the same gene structure as *SmABCG46*, *SmABCG35* was only expressed in the flowers (Table 1 and Additional file 1: Figure S1), which suggests that this gene might be involved in the transport of substances in the flowers of *S. miltiorrhiza*.

*SmABCG32* was a full-sized transporter and highly expressed in the leaves. Its homologous protein CrTPT2 in *Catharanthus roseus* is responsible for the transport of catharanthine [75], suggesting that *SmABCG32* might be involved in the transport of secondary metabolites in the leaves of *S. miltiorrhiza*. In addition, 6 half-sized ABCG transporters were expressed in various organs and showed higher expression levels in the flowers. For example, *SmABCG12* showed higher expression levels in the flowers compared to other tissues. SmABCG12’s homologue, AtABCG25, participates in the export of abscisic acid [61], indicating that SmABCG12 might be involved in the transport of plant hormones in the flowers of *S. miltiorrhiza*. *SmABCG19* was also highly expressed in the flowers and was homologous to AtABCG11 in *Arabisopsis* [66] and GhWBC1 in cotton [76], suggesting that SmABCG19 likely played roles in the transport substances that are related to the growth and development of *S. miltiorrhiza*. *SmABCG27* and *SmABCG28* showed the same expression patterns and were more highly expressed in the flowers compared to the rest of the plant. Both of SmABCG27 and SmABCG28 were half-sized proteins and were expressed in all organs except for the leaves (Table 1). Their homologue, AtABCG14, mediates the root-to-shoot translocation of *trans*-Zeatin in *Arabidopsis* [77]. Thus, SmABCG27 and SmABCG28 are likely involved in hormone transport in *S. miltiorrhiza. SmABCG15* was highly expressed in the leaves and also induced by MeJA (Table 1), indicating that *SmABCG15* might participate in the MeJA signal transduction pathway.

#### ABCI subfamily

**Fig. 5:**
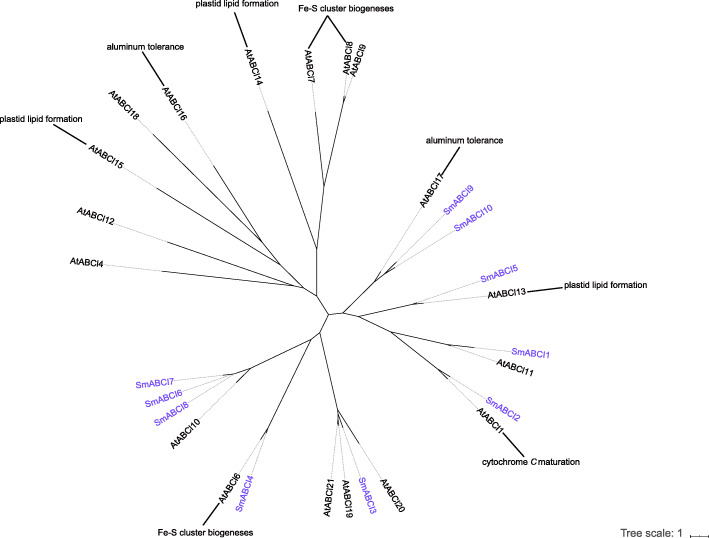
Phylogenetic tree of the ABCI subfamily. Phylogenetic analysis of ABCI proteins of *S. miltiorrhiza*, Arabidopsis and other plants

The *Arabidopsis* genome contains 15 ABCIs, whereas the rice genome contains 10 members of this subfamily [6, 12]. The ABCI subfamily of *S. miltiorrhiza* consisted of 10 genes (Fig. 5), all of which harboured only one soluble NBD. These ABCI transporters were expressed in all tissues in *S. miltiorrhiza* (Table 1). SmABCI4 might be involved in the biosynthesis of Fe/S clusters in the leaves because its expression profile was similar to its homologous gene *AtABCI6* [78]. SmABCI5 was a homologous to AtABCI13, and the latter is involved in the formation of plastid lipids [78]. SmABCI2 showed high similarity to AtABCI1, which is related to the maturation of cytochrome *c* [79].

### Gene expression profiling analysis

The gene expression profiles of the 114 putative ABC transporters were detected from the transcriptome data generated from the different organs (leaf, stem, flower, root) and tissues (periderm, phloem, xylem) of *S. miltiorrhiza* in our previous studies [23, 24] (Table 1). The relative expression levels of these genes were analysed by the FPKM values verified by transcriptome sequencing in our previous studies [23, 24]. According to the gene expression pattern, 13 genes (*SmABCB4*, *SmABCB7*, *SmABCC1*, *SmABCC5*, *SmABCD1*, *SmABCE1*, *SmABCF3-SmABCF6* and *SmABCG46*) were highly expressed in all organs. By contrast, 11 genes showed low expression levels in all organs, including *SmABCA3*, *SmABCB1*, *SmABCB3*, *SmABCC3*, *SmABCC6*, *SmABCC14*, *SmABCF2*, *SmABCG13*, *SmABCG33* and *SmABCI2*. Furthermore, a total of 46 genes were rarely expressed in all organs, including *SmABCB6*, *SmABCB8*, *SmABCB12*, *SmABCB15-SmABCB17*, *SmABCB20-SmABCB23*, *SmABCB25-SmABCB27*, *SmABCB29*, *SmABCB31*, *SmABCC7*, *SmABCC9*, *SmABCC12*, *SmABCD2*, *SmABCF1*, *SmABCG2*, *SmABCG3*, *SmABCG5-SmABCG7*, *SmABCG9*, *SmABCG10*, *SmABCG14*, *SmABCG16-SmABCG18*, *SmABCG20-SmABCG23*, *SmABCG25*, *SmABCG26*, *SmABCG29-SmABCG31*, *SmABCG34*, *SmABCG36*, *SmABCG38*, *SmABCG39*, *SmABCG41* and *SmABCI7*. The expression of some genes showed tissue- or organ-dependent specificity. For example, 14 genes were highly expressed in the roots and root tissues, including *SmABCA1*, *SmABCB2*, *SmABCB5*, *SmABCB9*, *SmABCB30*, *SmABCC2*, *SmABCC4*, *SmABCC11*, *SmABCC13*, *SmABCG1*, *SmABCG4*, *SmABCG11*, *SmABCG40* and *SmABCI5*. Although nine genes were expressed in the flowers, stems and leaves, they were not expressed in the roots and root tissues, such as *SmABCB14*, *SmABCB24*, *SmABCC10*, *SmABCG12*, *SmABCG15*, *SmABCG19*, *SmABCG32*, *SmABCG45* and *SmABCI6*. The 13 genes were expressed more highly in the flowers, stems, leaves and roots but not in the three tissues of the root, including *SmABCA2*, *SmABCB11*, *SmABCB19*, *SmABCC8*, *SmABCF7*, *SmABCG24*, *SmABCG27*, *SmABCG28*, *SmABCG37*, *SmABCG43*, *SmABCI1*, *SmABCI3* and *SmABCI8*. Moreover, 6 genes were highly expressed in the root rather than in other tissues, including *SmABCB10*, *SmABCB13*, *SmABCB18*, *SmABCB28*, *SmABCG8* and *SmABCG45*. The different expression profiles of these ABC genes suggested that they might perform different gene functions in *S. miltiorrhiza.*

### Verification of the gene expression of candidate transporters in the transport of tanshinone and salvianolic acid

**Fig. 6:**
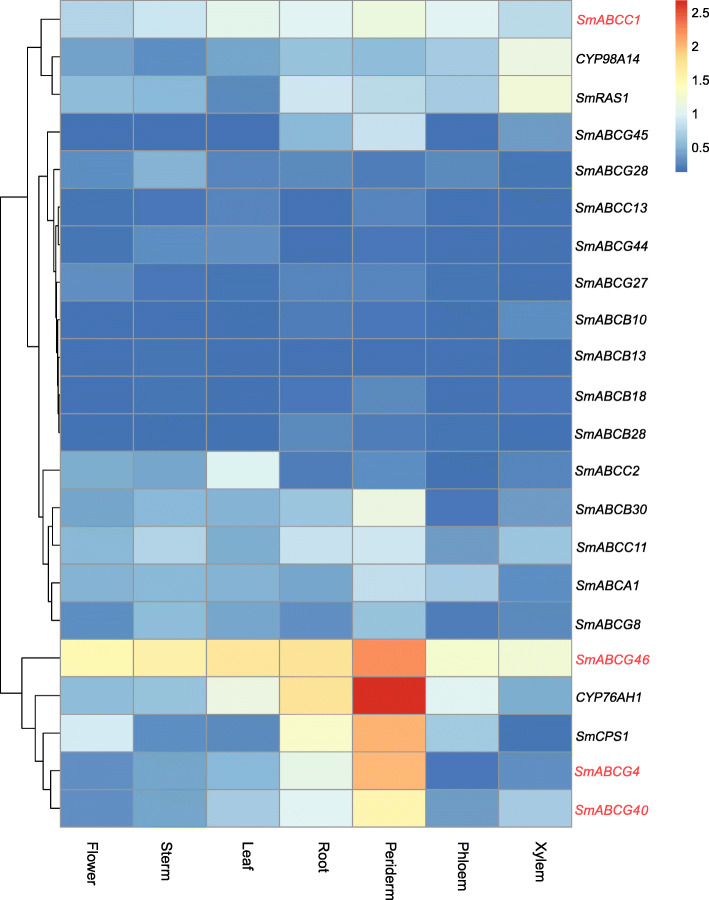
Co-expression profile analysis of candidate transporter genes and key enzyme genes involved in the biosynthetic pathway of tanshinone and salvianolic acid. The heat map showed the relative gene expression profiles of 18 candidate genes detected by qRT-PCR in the organs (root, stem, leaf and flower) and the tissues of the root, including the periderm, phloem and xylem. The gene co-expression patterns amongst the candidate genes with the key enzyme-coding genes involved in tanshinone biosynthesis (*CYP76AH1* and *SmCPS1*) and salvianolic acid biosynthesis (*CYP98A14* and *SmRAS1*) were calculated and clustered by a hierarchical method

The tissue-specific expression of some transporter genes might be related to their function in specific tissues or organs. In contrast, some genes showed indistinguishable expression profiles in all tissues, suggesting that they might play a role in the transport of basic substances and primary metabolites in all cells. Considering that tanshinone and SA were primarily synthesised and accumulated in the roots of *S. miltiorrhiza* [1–3, 24], we hypothesised that the highly abundant transporter genes expressed in the roots of *S. miltiorrhiza* might be related to the transportation of tanshinone and SA. Based on gene expression profiles and transcriptome analysis (Table 1), we identified out 18 candidate genes which were highly expressed in the roots of *S. miltiorrhiza* for qRT-PCR verification (Additional file 3: Figure S2). These 18 genes included members of the following subfamilies: 1 ABCA (*SmABCA1*), 5 ABCBs (*SmABCB10*, *SmABCB13*, *SmABCB18*, *SmABCB28* and *SmABCB30*), 4 ABCCs (*SmABCC1*, *SmABCC2*, *SmABCC11* and *SmABCC13*) and 8 ABCGs (*SmABCG8*, *SmABCG27*, *SmABCG28*, *SmABCG40*, *SmABCG44*, *SmABCG45* and *SmABCG46*). Amongst these candidate ABC genes, we found that the expression patterns of *SmABCG46*, *SmABCG40* and *SmABCG4* were nearly identical to that of *CYP76AH1* and *SmCPS1*, which are key enzyme genes involved in the biosynthetic pathway of tanshinone (Fig. 6). Moreover, *SmABCC1* was co-expressed with *CYP98A14* and *SmRAS*, which encode the key enzymes in the biosynthetic pathway of SA in *S. miltiorrhiza* (Fig. 6). Therefore, these four candidate ABC transporters which are co-expressed with key enzyme genes in the biosynthesis of tanshinone and SA likely participated in the intracellular transport of these two active compounds in *S. miltiorrhiza.* All the four candidate SmABCs were labelled with a red star in Figs. 3a and 4, respectively*.*

**Fig. 7:**
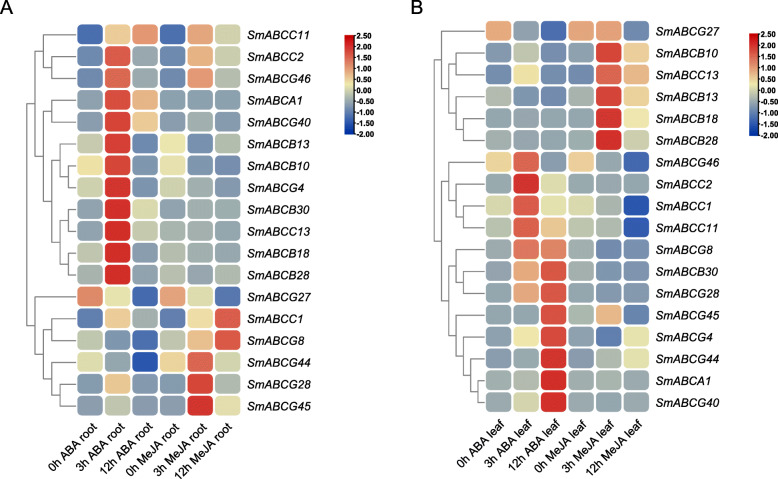
qRT-PCR detection of the expression profiles of the 18 selected genes induced by ABA and MeJA. Heat maps of the relative expression of 18 SmABCs under the treatment of ABA and MeJA. Scaled log2 expression values based on qRT-PCR data are shown from blue to red, indicating low to high expression. **a** The relative expression of these *SmABC*s in the root of 1-year old *S. miltiorrhiza* seedling under ABA (10 mM) and MeJA (200 μM) treatment. **b** The relative expression of these *SmABC*s in the leaves of 1-year old *S. miltiorrhiza* seedling under ABA (10 mM) and MeJA (200 μM) treatment

**Fig. 8:**
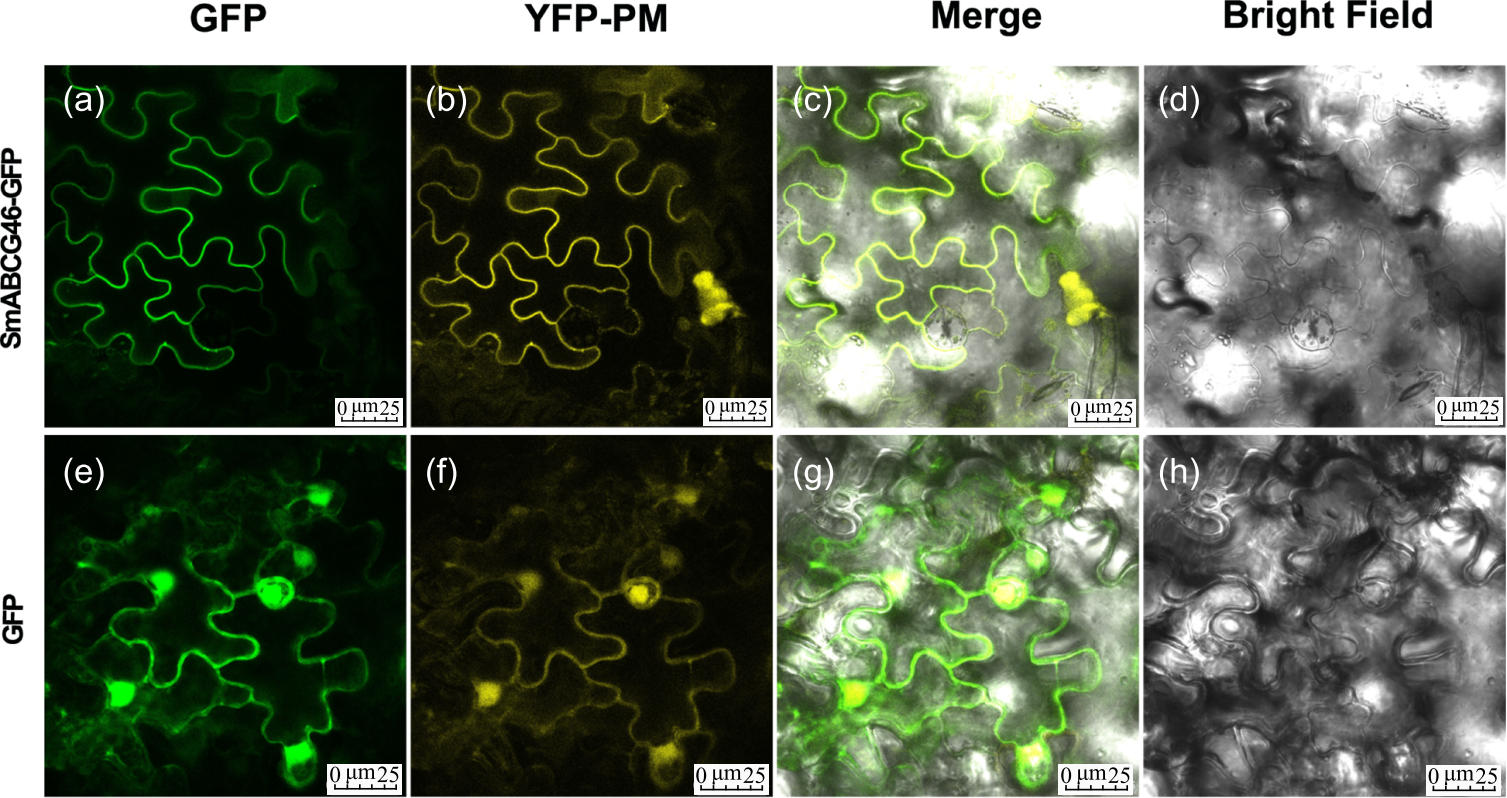
The subcellualr localization of SmABCG46. Confocal laser scanning microscopy images showed tabacco leaf epidermal cells transiently expressing either SmABCG46::GFP (**a**-**d**) or GFP (**e**-**h**) together with PM-YFP (plasma membrane marker, Chen et al. [80]). GFP was fused to a downstream of the CDS sequence corresponding to the SmABCG46 without stop codon and transiently expressed in tobacco leaf. GFP fluorescence indicates the location of fusion protein (shown in green). The location of plasma membrane was determined by the fluorescence of PM-YFP. Column labelled “Merged” represents all the combined fluorescent signals. **a**/**e** Confocal images via the GFP channel only. **b**/**f** Confocal images of the YFP fluorescence marking with PM position. **c**/**g** Merged images of GFP (green) and YFP (yellow) together with bright field. **d**/**h** Bright field. Scale bar, 25 μm. The measurement resolution of the acquired image was 1024 × 1024

In addition, the inducible expression profiles of these 18 candidate genes in the root of 1-year-old seedlings was explored using treatment with abscisic acid (ABA) and methyl jasmonate (MeJA) (Fig. 7). Under the induction of ABA treatment for 3 h, a total of 11 genes were strongly up-regulated in the roots of *S. miltiorrhiza*, and another 5 genes were significantly up-regulated in the roots induced by MeJA (Fig. 7a). In ABA-treated leaves of *S. miltiorrhiza*, totally 12 genes were induced and their expression was up-regulated, and another 5 genes were induced by MeJA and their expression was significantly up-regulated in the leaves (Fig. 7b). For the four candidate genes, the high of *SmABCG40* and *SmABCG4* was induced by 12 h of the ABA treatment in the leaves (Fig. 7b), while in the roots, the expression of *SmABCG46* and *SmABCC1* was significantly induced by 3 h of ABA treatment (Fig. 7a). Under MeJA treatment, the gene expression levels of *SmABCG46* and *SmABCC1* increased significantly at different time points in the root (Fig. 7a). In contrast, the expression of *SmABCG4* and *SmABCG44* was detected to be induced by MeJA treatment in the leaves (Fig. 7b). The expression pattern of these genes induced by MeJA in leaves is slightly different from the results of previous studies [23], which may be caused by different experimental materials and different treatment methods. These results indicated that *SmABCG46* and *SmABCC1* may be responsible for the transport of biologically active compounds in roots, while the other two candidate genes may have functions in the leaves of *S. miltiorrhiza*. Furthermore, compared with a plasma membrane located marker protein YFP-PM [80], the subcellular location of SmABCG46 is in the plasma membrane, which indicates that this ABC protein is involved in the process of transport across the plasma membrane (Fig. 8).

### Identification of *cis*-elements in SmABC gene

To identify the putative *cis*-elements in SmABC promoters, the 1500 bp DNA sequences upstream of the start codon (ATG) for each of the 114 SmABCs were screened using the Plant *Cis*-acting Regulatory DNA Elements (PLACE) (https://www.dna.affrc.go.jp/PLACE/). PLACE identified a total of 267 different *cis*-elements in the promoter of SmABC genes, and there were nine common *cis*-regulatory elements in all promoter regions of SmABC genes (Additional file 4: Table S2 and Table 2). Three common *cis*-regulatory elements WRKY71OS, ARR1AT and GT1CONSENSUS are known to play a role in the regulation of plant defences and plant hormones including GA, ABA, and SA. This suggests that these plant hormones could also affect the expression of SmABC genes. WRKY71OS is part of the plant response to pathogen-induced biotic stresses, which indicates that SmABC proteins might affect plant response to biotic stress in *S. miltiorrhiza*. Furthermore, the common cis-elements of GTGANTG10 and POLLEN1LELAT52 are required for transcriptional regulation in pollen, indicating that SmABC proteins may be involved in the reproductive processes of *S. miltiorrhiza*. Two common cis-regulatory elements, GATABOX and GT1CONSENSUS, are thought to be required for transcriptional regulation with light and therefore might affect plant growth and development. Out of the 9 common *cis*-regulatory elements, DOFCOREZM plays a role in carbon metabolism, suggesting that SmABCs are also likely to participate in energy metabolism.

**Table 2:** Putative *cis*-elements identified in the promoter sequences of SmABC genes

***Cis***-element	Signal sequence	SITE	Expression pattern
CACTFTPPCA1	YACT	S000449	C4 plant, mesophyll
DOFCOREZM	AAAG	S000265	Leaf, shoot, carbon metabolism
CAATBOX1	CAAT	S000028	Seed
ARR1AT	NGATT	S000454	Response regulator
GT1CONSENSUS	GRWAAW	S000198	Light, leaf, shoot, SA
GATABOX	GATA	S000039	Chlorophyll a/b binding protein, leaf, shoot
GTGANTG10	GTGA	S000378	Pollen
POLLEN1LELAT52	AGAAA	S000245	Pollen
WRKY71OS	TGAC	S000447	PR proteins, GA, ABA, plant defences

## Discussion

In plants, the first ABC protein was initially identified as a transporter involved in the final detoxification process [81]. Since this discovery, many reports have shown that the function of this type of transporter extends far beyond detoxification. In recent years, ABC transporters and have become a major focus for research in plants. This is not only due to their overall roles in a variety of processes, such as pathogen response, surface lipid deposition, accumulation of phytic acid in seeds, and the transport of plant hormones, but rather for the reason that they play essential roles in plant growth and development, response to abiotic stressors and in the interactions between plants and their environment.

In this study, a total of 114 ABC proteins were identified in the genome of *S. miltiorrhiza*, amongst which, 86 members encoded for ABC transporters with TMDs, including 50 full-sized ABC transporters. The ratio of ABC proteins to full-sized ABC transporters in *S. miltiorrhiza* was similar to that in *Arabidopsis* [6, 12]. The total number of genes encoding for ABC proteins was nearly identical in the two species, despite of the large differences in genome size (615 Mb versus 125 Mb) and gene content (30,478 versus 25,498 genes) [12, 21]. The identification of *S. miltiorrhiza* ABC proteins and their comparative analysis with the *Arabidopsis* ABC transporters revealed strong evidence of conservation of ABC transporters between the two species. A single plant species can synthesize thousands of different molecules, and these molecules can be transported across the plasma membrane of one or more organelles, which might explain the large size of the ABC transporter gene family in plants compared to other organisms [82].

On the basis of phylogenetic analysis, except for ABCH, the *S. miltiorrhiza* ABC proteins were divided into subfamilies from ABCA to ABCI. The ABCG (46 genes), ABCB (31 genes) and ABCC (14 genes) subfamily have the most members, whiles the ABCA, ABCD and ABCE subfamily have fewer members (Table 1). These relative abundances were similar to the subfamily distribution of *Z. mays* [14], *A. comosus* [16], *L. japonicus* [20], and *O. sativa* [83] (Additional file 5: Table S3). In these species, the number of ABC genes that have identified ranged from 91 to 314, including 137 members in *Amborella trichopoda* [83], 100 members in *A. comosus* [16], 132 members in *A. lyrata* [83]*,* 130 members in *A. thaliana* [6], 138 members in *Brachypodium distachyon* [83], 314 members in *B. napus* [15], 179 members in *B. rapa* [83], 200 members in *C. annuum* [18], 185 members in *C. baccatum* [18], 187 members in *C. chinense* [18], 113 members in *Carica papaya* [83], 271 members in *Glycine max* [83], 91 members in *L. japonicas* [20], 141 members [83] and 127 members [82] in *O. sativa*, 204 members in *Populus trichocarpa* [83], 154 members in *S. lycopersicum* [17], 181 members in *V. vinifera* [83], and 130 members in *Z. mays* [14] (Additional file 5: Table S3). Among angiosperms, the subfamilies ABCG, ABCB, and ABCC are the most abundant, while the subfamilies ABCD and ABCE have the least members. For the ABCE subfamily, only one member was identified in *S. miltiorrhiza*. The members of most subfamilies, except for the ABCI subfamily, grouped more closely with each other than with members of other subfamilies (Fig. 1). Similarly, some members of ABCI also did not clustered with a group with high homology in *Arabidopsis* [6].

In *Arabidopsis*, various subfamilies of ABC transporters contain different conserved domains and perform various biological functions. Similar to *Arabidopsis* [6] and grape [13], only one full-sized ABC transporter (SmABCA1) had the longest gene sequence in the *S. miltiorrhiza* genome, belonging to the ABCA subfamily, and homologous to AtABCA1 (Table 1 and Fig. 2a), which implied the function of SmABCA1 maybe similar to AtABCA1. Phylogenetic analyses revealed that SmABCA3 clustered closest to AtABCA11in Arabidopsis, while SmABCA2 clustered with the group containing AtABCA10 (Fig. 2a), indicating that these two genes may be involved in the stress response of *S. miltiorrhiza.* In *Arabidopsis*, a set of ABCB proteins (e.g. the AtABCB1, AtABCB4, AtABCB14, AtABCB15, AtABCB19 and AtABCB21) have been reported to be involved in polar auxin transport as their mutants show reduced auxin transport [35, 40, 41, 84, 85]. This led us to speculate that the functions of these genes in *S. miltiorrhiza* (such as SmABCB10–13, SmABCB19, SmABCB29–31) may be homologous to those in *Arabidopsis* ABCB and thus might be involved in plant hormone transport (Fig. 2b). AtABCC1 and AtABCC2 contributed to the tolerance of Arabidopsis to admium (II) and mercury (II), while in the absence of AtABCC2, AtABCC1 conferred great tolerance to divalent heavy metals [86]. Similarly, AtABCC6 [52] might also enhance improve plant heavy metal tolerance. In addition, vacuolar membrane-localized ABC transporters, such as AtABCC4 [51], regulate the concentration of folate in the cytoplasm by transporting excess folic acid to the vacuole, indicating that ABCC transporters are also important for folic acid storage. The knockout mutants of Arabidopsis AtABCC5 exhibited a low phytate phenotype [44]. These results indicated that ABCC transporters play important roles in the transportation of primary products and in improving the heavy metal tolerance in plants. These findings provide valuable information for further exploration of the ABCC genes in *S. miltiorrhiza*, such as SmABCC1, SmABCC6, SmABCC5, SmABCC11 and SmABCC12 (Fig. 3a).

Similar to other plants, ABCG was the largest ABC transporter in *S. miltiorrhiza* (Table 1 and Fig. 4)*.* Several members of the ABCG subfamily in Arabidopsis, such as AtABCG25, AtABCG30, AtABCG31, and AtABCG40 were high affinity ABA transporters [60, 61], while AtABCG14 participated in transport of cytokinin [77]. AtABCG36 regulated the sensitivity of plants to the auxin precursor indole-3-butyric acid [87]. Furthermore the AtABCG37 participated in the secretion of scopoletin and scopoletin derivatives by Arabidopsis roots in response to iron deficiency [88]. Lr34 was involved in the resistance of wheat to various fungal pathogens [89], while CsPDR8 and CsPDR12 were related to the hormone response of cucumber [90]. StPDR2 [91] and OsPDR9 [92] conferred resistance to the biotic and abiotic stresses in tomato and in rice, respectively, and PhPDR2 was identified as a petuniasterone transporter in leaves and trichomes of *Petunia hybrida* [93]. NbABCG1/2 was involved in the export of antimicrobial diterpenes and capsidiol for defence against *Phytophthora infestans* [94], and *NtPDR3* in *N. tabacum* was induced to express iron deficiency in the culture medium [95]. The function of AtABCG genes identified in Arabidopsis are sufficient to demonstrate the diversity of gene functions in the ABCG subfamily [96]. It was worth noting that several members of the ABCG subfamily also participated in pathogen defense and/or the crosstalk between plants and microorganisms, with secondary metabolite-dependent processes. In addition, the tanshinone and SA are also secondary metabolites with diverse pharmacological activities in *S. miltiorrhiza*. Some members of the ABCG subfamily may participate in the transport of these active compounds in this medicinal plant.

Gene expression profiles are complex phenotypic datasets that can reflect the biological processes of target genes involved in metabolism, tissue, organ development and differentiation, and response to environmental changes in plants. In this study, we analyzed a subset gene expression profiles in several organs/tissues of *S. miltiorrhiza*. Since the genes in the same biosynthetic pathway are generally co-expressed, we compared the expression patterns of all 18 candidate ABC transporter genes with the upstream genes encoding SmCPS1, CYP76AH1, RAS and CYP98A14, which are key enzymes involved in tanshinone and SA biosynthesis, respectively (Fig. 6 and Additional file 3: Figure S2). This co-expression analysis further suggested that three ABCG members (*SmABCG46*, *SmABCG40* and *SmABCG4*) and one ABCC member (*SmABCC1*) members might be involved in transport of tanshinone and SA in *S. miltiorrhiza*, respectively (Fig. 6)*.* The co-expression of the transporter genes with the key enzymatic genes in the secondary metabolic pathway (Fig. 6) and the expression induced by ABA and MeJA (Fig. 7) provided evidence that these transporters might be involved in the transport of secondary metabolites in *S. miltiorrhiza*. For example, *CsABCC4a* and *CsABCC2*, highly expressed in the stigmas of *C. sativus,* enabled crocin transport in yeast microsomes and were highly co-expressed with total crocin levels and/or *CsCCD2*, which was the first described enzyme in the crocin biosynthetic pathway [50]. ABCG14 was highly co-expressed with cytokinin biosynthesis and was the major root-to-shoot cytokinin transporter [77]. We anticipate that a functional study in the near future will elucidate the molecular and physiological functions of the lead candidate ABC transporter involved in tanshinone and SA transport in this important medicinal plant.

In addition, we found and confirmed the existence of tissue-specific ABC transporter gene expression profiles, which demonstrates the role of ABC transporter analysis to predictive tissue-dependent functions in *S. miltiorrhiza* and possible in other plants (Table 1, Figs. 6, and 7). These results provided not only valuable information for investigating the functions of the ABC transporter gene in *S. miltiorrhiza* but also an applied methodology for identifying, screening and validating candidate genes involved in bioactive secondary metabolite transport in medicinal plants based on genome and transcriptome datasets.

## Conclusion

In this study, we identified and analysed ABC transporters in *S. miltiorrhiza* for the first time and provided the fundamental and detailed information about *S. miltiorrhiza* ABC proteins. The information included all the ABC proteins in *S. miltiorrhiza* with the gene name, domain topology, gene expression profiles and phylogenetic trees of subfamily members and orthologues in other plants, showing the reported physiological functions. Based on the previous studies on the functions of ABC genes, the functions of some ABC transporters with domain or expression characteristics were hypothesised in *S. miltiorrhiza*. Combined phylogenetic and co-expression analyses identified three genes (*SmABCG46*, *SmABCG40* and *SmABCG4*) and one ABCC member (*SmABCC1*) to be the lead candidates involved in tanshinone and SA transport, respectively. The transporters identified in the ABCG and ABCC subfamilies might be involved in the transport of secondary metabolites of *S. miltiorrhiza*. In addition, the transporters might be involved in the transport of anthocyanins, auxin and metal resistance have been identified in several ABC subfamilies of *S. miltiorrhiza*. Our study outlined the ABC proteins in the *S. miltiorrhiza* genome and explained their possible transporting pathways for some compounds, laying an important foundation for further research on the metabolic regulation, synthetic biology and utilisation of these compounds in *S. miltiorrhiza*. Our analysis provides new insight into the diversity and the predicted function of the entire ABC transporters in *S. miltiorrhiza* compared with *Arabidopsis*. These results will provide new insights into the function of ABC transporters in *S. miltiorrhiza.*

## Methods

### Plant materials and treatment

*S. miltiorrhiza* Bunge (line 99–3) was collected from the garden at the Institute of Medicinal Plant Development (IMPLAD) in Beijing. The plants were authenticated by Professor Yulin Lin of the IMPLAD using the morphological identification approach of the Flora of China. The 1-year-old *S. miltiorrhiza* seedlings were cultured in Hoagland basal salt medium (Coolaber, Beijing, China) (Catalog No. NSP1020) for 7 days, and then transferred to Hoagland medium containing ABA (10 mM) or MeJA (200 μM) for induction induction of 0 h (CK), 3 h and 12 h, respectively. *N. benthamiana* was grown in pots at 23 ± 2 °C under 16 h light/8 h dark photoperiod.

### Identification of ABC transporter genes in the *S. miltiorrhiza* genome

BLAST was used to align all the proteins in the *S. miltiorrhiza* genome with the ABC proteins in the *Arabidopsis* TAIR11 database (*E* value is less than 1e-5) and identify ABC homologues in *S. miltiorrhiza*. HMMER (https://www.ebi.ac.uk/Tools/hmmer/) was used to characterise the topology of ABC proteins containing TMD and NBD domains. The TMD domain in these ABC proteins was identified by PF12698, PF06472 and PF00664, and the NBD domain was identified by PF00005 in Pfam database (https://pfam.xfam.org/). A protein with at least one NBD domain was predicted to encode an ABC transporter. The domain analysis also provided evidence for the subfamily classification of the ABC family in *S. miltiorrhiza*. The obtained *S. miltiorrhiza* ABC protein sequences were submitted to the MEME Web server (http://meme-suite.org/) to confirm the relationship between the conserved motifs and genes.

### Phylogenetic analyses

Clustal X (http://www.clustal.org/) was used to perform sequence alignment on the deduced amino acid sequence of *S. miltiorrhiza* ABC proteins, and then, MEGA 6 was used to construct a phylogenetic tree with 1000 bootstrap repeats through the neighbor-joining method. The maximum likelihood method was used to establish phylogenetic trees of *S. miltiorrhiza* ABC transporter subfamilies with the ABC transporters that have been functionally identified in *Arabidopsis* and other plants (listed in Additional file 2 Table S1- sheet 1) to predict the function of these transporters in *S. miltiorrhiza*. Phylogenetic trees were embellished using the interactive Tree Of Life Platform (https://itol.embl.de/).

### Analysis of gene expression profiles using transcriptome data

*S. miltiorrhiza* (line 99–3) plants were grown in the medicinal plant garden of the Institute of Medicinal Plant Development. The transcriptome of different organs (flower, stem, leaf, root), root tissues (periderm, phloem, xylem) and leaves (with and without MeJA treatment) was sequenced by Illumina Hiseq 2000 technology in our previous studies [23, 24]. The expression profiles of 114 putative *S. miltiorrhiza* ABC genes were analysed with these transcriptome data. The candidate genes that were highly expressed in the roots of *S. miltiorrhiza* were selected for quantitative reverse transcription polymerase chain reaction (qRT-PCR) verification.

### qRT-PCR verification of gene expression profiles

The relative expression levels of 18 selected SmABC genes in different organs/tissues of *S. miltiorrhiza* and the expression patterns of these genes in the seedlings treated with ABA or MeJA were analysed by qRT-PCR. Total RNA was extracted from the flowers, stems, leave, roots and the root tissues including of periderm, phloem and xylem, all of which were isolated from the 2-year-old *S. miltiorrhiza*, and the roots/leave of 1-year-old seedlings treated with ABA (10 mM) or MeJA (200 μM) for 0 h (CK), 3 h and 12 h, according to the manufacturer’s instructions of the RNAprep Pure Plant Kit (TIANGEN, China), and then reversed into cDNA using the Promega GoScript Reverse Transcription System (Promega, Beijing, China). qRT-PCR was performed on an ABI PRISM 7500 real-time PCR system (Applied Biosys) using SYBR Premix Ex Taq™ (Takara, Beijing, China) with the following program: 95 °C for 30 s, 1 cycle; 95 °C for 5 s and 60 °C for 34 s, 40 cycles. The relative gene expression level was calculated using the 2^−ΔΔCT^ method [97] with the *SmActin* gene (Genbank number HM231319.1) as an internal reference. The experiments were performed in three independent biological experiments with three technical replicates. qRT-PCR was performed to determine the relative expression levels of the 18 candidate ABC genes along with *CYP98A14*, *SmRAS1*, *CYP76AH1* and *SmCPS*1, which were used as positive genes to calculate the correlation coefficient of co-expression for these ABC genes with the key enzyme-encoding genes involved in the biosynthesis of SAs and tanshinones. Tbtools [98] software was used to perform heat map analysis of gene expression induced by ABA and MeJA. The primers used in qRT-PCR are listed in Table S4 of Additional File 6.

The candidate gene of *SmABCG46* was cloned from *S. miltiorrhiza* using total RNA isolated from seedlings as a template for amplification. In order to perform subcellular localization analysis, the ORF of *SmABCG46* was introduced into the pCAMBIA1300-Super-GFP vector using the Seamless Cloning and Assembly Kit (Vazyme, Nanjing, China) according to the manufacturer’s instructions. The full-length coding region of *SmABCG46* (without stop codon) was fused with green fluorescent protein (GFP) in pCAMBIA1302 vector, and identified by sequencing. The expression vector was transiently introduced into *Agrobacterium* strain *GV3101*, and infiltrated into the leaves of *N. benthamiana*. After 48 or 72 h of infiltration, the GFP fluorescence of the gene was observed using a confocal laser scanning microscope (LEICA TCS SP8, Germany). The acquisition software is LAS AF Lite 3.0. The pCAMBIA1300-Super plasmid was transformed into tobacco leaves as a positive control. The location of plasma membrane was determined by the fluorescence of YFP-PM [80].

#### *Cis*-elements analysis

All the promoter sequences (1500 bp upstream of start codon “ATG”) of the SmABC transporters were extracted from the draft genome of *S. miltiorrhiza* [21] according to the Generic File Format (GFF) file. Then, the *cis*-elements of promoters for each gene were identified by PLACE Web Signal Scan-PLACE (https://www.dna.affrc.go.jp/PLACE/).

## Supplementary Information

**Additional file 1: Figure S1.** Conserved motifs of SmABC proteins. The motif in the SmABC proteins was identified by using Multiple Em for Motif Elicitation (MEME). Ten conserved motifs were identified and displayed in different colors

**Additional file 2: Table S1.** The ABC transporters that have been functionally identified from other plants used for phylogenetic tree analysis in this study (sheet 1) and the accession numbers of SmABCs deposited in the GenBank (sheet 2)

**Additional file 3: Figure S2.** qRT-PCR validation of the 18 selected candidate transporter genes involved in bioactive compound transportation in *S. miltiorrhiza. SmActin* was used as an internal control. Each gene has three biological replicates and three technical replicates

**Additional file 4: Table S2.** The *cis*-elements identified in the promoter sequences of all the SmABC genes

**Additional file 5: Table S3.** Comparative analysis of ABC proteins between *S. miltiorrhiza* and other plant species

**Additional file 6: Table S4.** Primers used in this study

## Data Availability

The datasets supporting the conclusions of this article are included with in the article and its additional files. The relative expression analysis from RNA-seq data and SMRT sequencing data of four different organs (root, stem, leaf, and flower) and three root tissues (periderm, phloem and xylem) as well the data from MeJA-treated leaves (200 μM) were derived from our previous studies [23, 24]. All the data have been submitted to the Sequence Read Archive (SRA) of the National Center for Biotechnology Information (NCBI) under accession numbers SRX753381, SRR1640458, SRP028388 and SRP051564. The accession numbers (MW890146 - MW890259) assigned to 114 SmABC genes in GenBank have been listed in Additional file 2 Table S1- sheet 2.

## Abbreviations

ABA: Abscisic acid
ABC: ATP-binding cassette
AOH: ABC one homolog
ATH: ABC two homolog
ATM: ABC transporter of the mitochondrion
4C-DHPL: 4-coumaroyl-3′,4′-dihydroxyphenyllactic acid
CPP: Copalyl diphosphate
CPS: Copalyl diphosphate synthase
CYP450: Cytochrome P450 monooxygenase
DHPL: 3,4-dihydroxyphenyllactic acid
GCN: General control non-repressible
MeJA: Methyl jasmonate
MDR: Multidrug resistance
MRP: Multidrug resistance-related protein
NAP: Non-intrinsic ABC protein
NBD: Nucleotide binding domain
OPDA: 12-oxophytodienoic acid
PDR: Pleiotropic drug resistance
PGP: P-glycoprotein
PMP: Peroxisomal membrane protein
RAS: Rosmarinic acid synthase
RNAi: RNA interference
RLI: RNase L inhibitor
SA: Salvianolic acid
SMC: Structural maintenance of chromosomes
TAP: Transporters associated with antigen processing
TMD: Transmembrane domain
WBC: White-brown complex homolog
