# Supplementary material for: Genome-wide analysis of ATP-binding cassette transporter provides insight to genes related to bioactive metabolite transportation in Salvia miltiorrhiza
Source: BMC Genomics. 2021 May 1;22:315. doi: 10.1186/s12864-021-07623-0 (PMC8088630; doi:10.1186/s12864-021-07623-0)
Supplement: Supplementary file 1 — Additional file 1: Figure S1. Conserved motifs of SmABC proteins. The motif in the SmABC proteins was identified by using Multiple Em for Motif Elicitation (MEME). Ten conserved motifs were identified and displayed in different colors [file 12864_2021_7623_MOESM1_ESM.pdf]

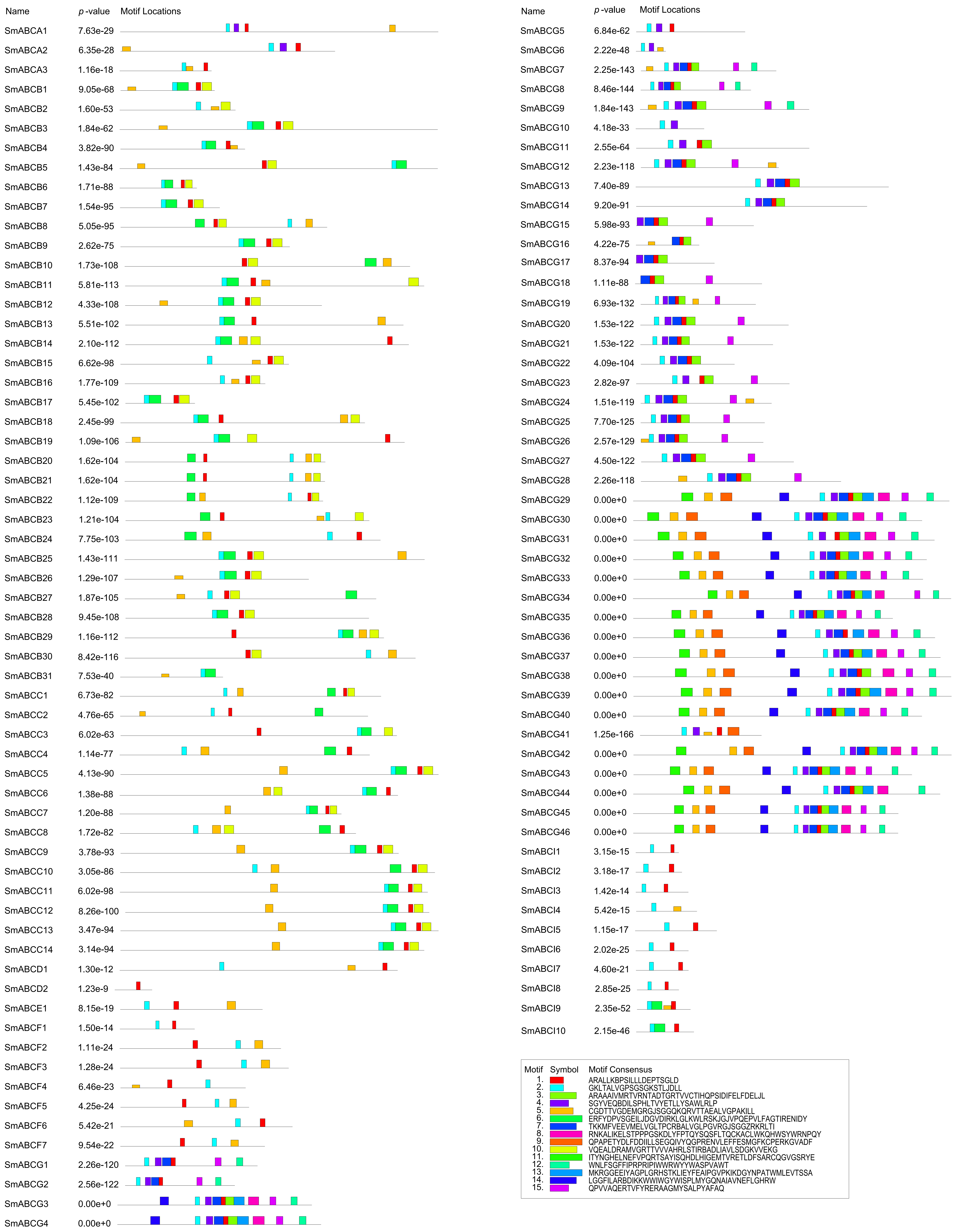
Name p-value Motif Locations
SmABCA1 7.63e-29
SmABCA2 6.35e-28
SmABCA3 1.16e-18
SmABCB1 9.05e-68
SmABCB2 1.60e-53
SmABCB3 1.84e-62
SmABCB4 3.82e-90
SmABCB5 1.43e-84
SmABCB6 1.71e-88
SmABCB7 1.54e-95
SmABCB8 5.05e-95
SmABCB9 2.62e-75
SmABCB10 1.73e-108
SmABCB11 5.81e-113
SmABCB12 4.33e-108
SmABCB13 5.51e-102
SmABCB14 2.10e-112
SmABCB15 6.62e-98
SmABCB16 1.77e-109
SmABCB17 5.45e-102
SmABCB18 2.45e-99
SmABCB19 1.09e-106
SmABCB20 1.62e-104
SmABCB21 1.62e-104
SmABCB22 1.12e-109
SmABCB23 1.21e-104
SmABCB24 7.75e-103
SmABCB25 1.43e-111
SmABCB26 1.29e-107
SmABCB27 1.87e-105
SmABCB28 9.45e-108
SmABCB29 1.16e-112
SmABCB30 8.42e-116
SmABCB31 7.53e-40
SmABCC1 6.73e-82
SmABCC2 4.76e-65
SmABCC3 6.02e-63
SmABCC4 1.14e-77
SmABCC5 4.13e-90
SmABCC6 1.38e-88
SmABCC7 1.20e-88
SmABCC8 1.72e-82
SmABCC9 3.78e-93
SmABCC10 3.05e-86
SmABCC11 6.02e-98
SmABCC12 8.26e-100
SmABCC13 3.47e-94
SmABCC14 3.14e-94
SmABCD1 1.30e-12
SmABCD2 1.23e-9
SmABCE1 8.15e-19
SmABCF1 1.50e-14
SmABCF2 1.11e-24
SmABCF3 1.28e-24
SmABCF4 6.46e-23
SmABCF5 4.25e-24
SmABCF6 5.42e-21
SmABCF7 9.54e-22
SmABCG1 2.26e-120
SmABCG2 2.56e-122
SmABCG3 0.00e+0
SmABCG4 0.00e+0
Name p-value Motif Locations
SmABCG5 6.84e-62
SmABCG6 2.22e-48
SmABCG7 2.25e-143
SmABCG8 8.46e-144
SmABCG9 1.84e-143
SmABCG10 4.18e-33
SmABCG11 2.55e-64
SmABCG12 2.23e-118
SmABCG13 7.40e-89
SmABCG14 9.20e-91
SmABCG15 5.98e-93
SmABCG16 4.22e-75
SmABCG17 8.37e-94
SmABCG18 1.11e-88
SmABCG19 6.93e-132
SmABCG20 1.53e-122
SmABCG21 1.53e-122
SmABCG22 4.09e-104
SmABCG23 2.82e-97
SmABCG24 1.51e-119
SmABCG25 7.70e-125
SmABCG26 2.57e-129
SmABCG27 4.50e-122
SmABCG28 2.26e-118
SmABCG29 0.00e+0
SmABCG30 0.00e+0
SmABCG31 0.00e+0
SmABCG32 0.00e+0
SmABCG33 0.00e+0
SmABCG34 0.00e+0
SmABCG35 0.00e+0
SmABCG36 0.00e+0
SmABCG37 0.00e+0
SmABCG38 0.00e+0
SmABCG39 0.00e+0
SmABCG40 0.00e+0
SmABCG41 1.25e-166
SmABCG42 0.00e+0
SmABCG43 0.00e+0
SmABCG44 0.00e+0
SmABCG45 0.00e+0
SmABCG46 0.00e+0
SmABCI1 3.15e-15
SmABCI2 3.18e-17
SmABCI3 1.42e-14
SmABCI4 5.42e-15
SmABCI5 1.15e-17
SmABCI6 2.02e-25
SmABCI7 4.60e-21
SmABCI8 2.85e-25
SmABCI9 2.35e-52
SmABCI10 2.15e-46
Motif Symbol Motif Consensus
1. ARALLKBPSILLLDEPTSGLD
2. GKLTALVGPSGSGKSTLJDLL
3. ARAAAIVMRTVRNTADTGRTVVCTIHQPSIDIFELFDELJL
4. SGYVEQBDILSPHLTVYETLLYSAWLRLP
5. CGDTTVGDEMGRGJSGGQKQRVTTAEALVGPAKILL
6. ERFYDPVSGEILJDGVDIRKLGLKWLRSKJGJVPQEPVLFAGTIRENIDY
7. TKKMFVEEVMELVGLTPCRBALVGLPGVRGJSGGZRKRLTI
8. RNKALIKELSTPPPGSKDLYFPTQYSQSFLTQCKACLWKQHWSYWRNPQY
9. QPAPETYDLFDDIILLSEGQIVYQGPRENVLEFFESMGFKCPERKGVADF
10. VQEALDRAMVGRTTVVVAHRLSTIRBADLIAVLSDGKVVEKG
11. ITYNGHELNEFVPQRTSAYISQHDLHIGEMTVRETLDFSARCQGVGSRYE
12. WNLFSGFFIPRPRIPIWWRWYYWASPVAWT
13. MKRGGEEIYAGPLGRHSTKLIEYFEAIPGVPKIKDGYNPATWMLEVTSSA
14. LGGFILARBDIKKWWIWGYWISPLMYGQNAIAVNEFLGHRW
15. QPVVAQERTVFYRERAAGMYSALPYAFAQ
