# Supplementary material for: Genome-wide analysis of ATP-binding cassette transporter provides insight to genes related to bioactive metabolite transportation in Salvia miltiorrhiza
Source: BMC Genomics. 2021 May 1;22:315. doi: 10.1186/s12864-021-07623-0 (PMC8088630; doi:10.1186/s12864-021-07623-0)
Supplement: Supplementary file 3 — Additional file 3: Figure S2. qRT-PCR validation of the 18 selected candidate transporter genes involved in bioactive compound transportation in S. miltiorrhiza. SmActin was used as an internal control. Each gene has three biological replicates and three technical replicates [file 12864_2021_7623_MOESM3_ESM.pdf]

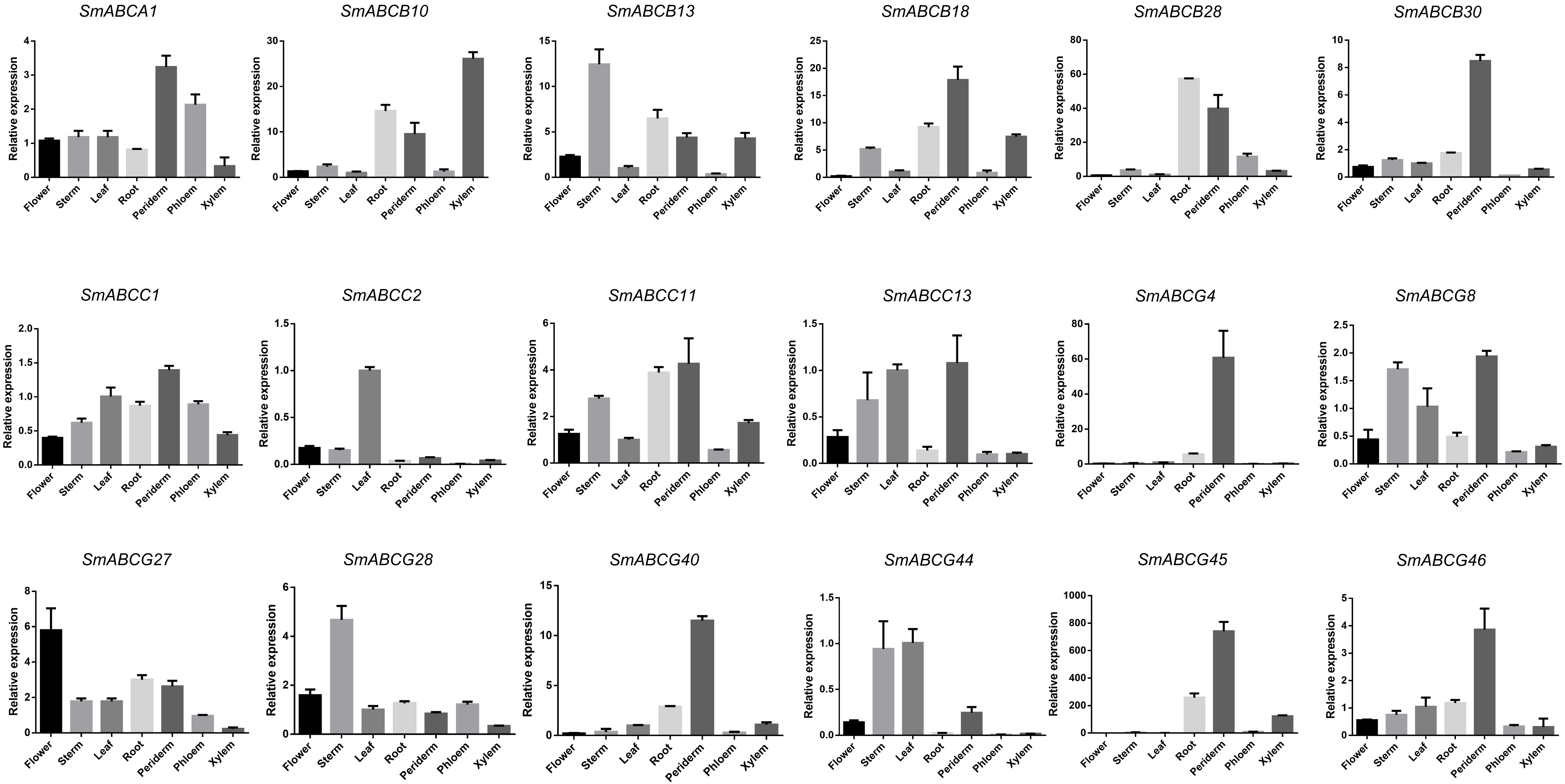

SmABCA1
SmABCB10
SmABCB13
SmABCB18
SmABCB28
SmABCB30
SmABCC1
SmABCC2
SmABCC11
SmABCC13
SmABCG4
SmABCG8
SmABCG27
SmABCG28
SmABCG40
SmABCG44
SmABCG45
SmABCG46
Relative expression
Flower
Sterm
Leaf
Root
Periderm
Phloem
Xylem
0
1
2
3
4
5
6
8
10
15
20
25
30
40
60
80
0.0
0.5
1.0
1.5
2.0
2.5
200
400
600
800
1000
