# Supplementary material for: Genome-wide analysis of ATP-binding cassette transporter provides insight to genes related to bioactive metabolite transportation in Salvia miltiorrhiza
Source: BMC Genomics. 2021 May 1;22:315. doi: 10.1186/s12864-021-07623-0 (PMC8088630; doi:10.1186/s12864-021-07623-0)
Supplement: Supplementary file 5 — Additional file 5: Table S3. Comparative analysis of ABC proteins between S. miltiorrhiza and other plant species [file 12864_2021_7623_MOESM5_ESM.docx]

**Additional file 5 Table S3 Comparative analysis of ABC proteins between *S. miltiorrhiza* and other plant species**

| **Species** | **Total**  **No.** | **ABC transporter subfamilies** | | | | | | | | **Ref** |
| --- | --- | --- | --- | --- | --- | --- | --- | --- | --- | --- |
|  |  | **ABCA** | **ABCB** | **ABCC** | **ABCD** | **ABCE** | **ABCF** | **ABCG** | **ABCI** |  |
| *Amborella trichopoda* | **137** | 4 | 19 | 17 | 2 | 2 | 5 | 46 | 42 | 83 |
| *Ananas comosus* | **100** | 5 | 20 | 16 | 2 | 1 | 5 | 42 | 9 | 16 |
| *Arabidopsis lyrata* | **132** | 12 | 25 | 15 | 2 | 3 | 10 | 43 | 22 | 83 |
| *Arabidopsis thaliana* | **130** | 12 | 29 | 15 | 2 | 3 | 5 | 43 | 21 | 6 |
| *Brachypodium distachyon* | **138** | 6 | 32 | 19 | 4 | 4 | 7 | 44 | 22 | 83 |
| *Brassica napus* | **314** | 30 | 69 | 47 | 5 | 13 | 14 | 116 | 20 | 15 |
| *Brassica rapa* | **179** | 11 | 38 | 21 | 2 | 7 | 7 | 63 | 30 | 83 |
| *Capsicum annuum* | **200** | 10 | 48 | 26 | 2 | 1 | 10 | 95 | 8 | 18 |
| *Capsicum baccatum* | **185** | 7 | 41 | 24 | 3 | 1 | 6 | 94 | 9 | 18 |
| *Capsicum chinense* | **187** | 9 | 44 | 23 | 5 | 1 | 6 | 91 | 8 | 18 |
| *Carica papaya* | **113** | 5 | 18 | 13 | 3 | 2 | 4 | 36 | 32 | 83 |
| *Glycine max* | **271** | 8 | 49 | 40 | 10 | 2 | 10 | 113 | 39 | 83 |
| *Lotus japonicus* | **91** | 3 | 15 | 17 | 2 | 1 | 6 | 36 | 11 | 20 |
| *Oryza sativa* | **141** | 6 | 28 | 17 | 3 | 1 | 6 | 56 | 24 | 83 |
| *Oryza sativa* | **127** | 6 | 27 | 17 | 3 | 2 | 6 | 50 | 16 | 82 |
| *Populus trichocarpa* | **204** | 6 | 40 | 29 | 3 | 2 | 4 | 78 | 42 | 83 |
| *Salvia miltiorrhiza* | **114** | 3 | 31 | 14 | 2 | 1 | 7 | 46 | 10 | **This study** |
| *Solanum lycopersicum* | **154** | 9 | 29 | 26 | 2 | 2 | 6 | 70 | 10 | 17 |
| *Vitis vinifera* | **181** | 5 | 30 | 26 | 1 | 1 | 6 | 71 | 41 | 83 |
| *Zea mays* | **130** | 6 | 31 | 13 | 4 | 2 | 7 | 54 | 13 | 14 |
